# New susceptibility alleles associated with severe coronary artery stenosis in the Lebanese population

**DOI:** 10.1186/s12920-021-00942-x

**Published:** 2021-03-25

**Authors:** Victor Wakim, Elie Abi Khalil, Angelique K. Salloum, Georges Khazen, Michella Ghassibe-Sabbagh, Pierre A. Zalloua

**Affiliations:** 1 School of Medicine, Lebanese American University, Beirut, Lebanon; 2 Department of Natural Sciences, School of Arts and Sciences, Lebanese American University, Beirut, Lebanon; 3 Harvard School of Public Health, Boston, MA 02215 USA

**Keywords:** Coronary artery disease, Lebanese population, GWAS, *PCSK6*, *TSPAN14*

## Abstract

**Background:**

Coronary Artery Disease (CAD) is the narrowing or blockage of the coronary arteries. It is closely associated with numerous genetics and environmental factors that have been extensively evaluated in various populations. In recent studies, severe phenotypes have been strongly linked to genetic risk factors.

**Methods:**

This study investigated the association of clinical, demographic, and genetic factors with severe coronary artery stenosis phenotypes in our population composed of 1734 individuals with severe coronary stenosis (≥ 50% in coronary vessels) and comparing them to 757 controls with no evidence of stenosis on angiography. We performed generalized linear model (GLM) genome-wide association studies to evaluate three stratification models and their associations to characteristics of the clinical disease. In model 1, patients were not stratified. In model 2, patients were stratified based on presence or absence of CAD family history (FxCAD). In model 3, patients were stratified by young age of CAD onset.

**Results:**

Eight SNPs (single nucleotide polymorphism) were significantly associated with severe CAD phenotypes in the various models $\left( {p < 5 \times 10^{ - 7} } \right)$, four of these SNPs were associated with severe CAD and the four others were specifically significant for young CAD patients. While these SNPs were not previously reported for association with CAD, six of them are present in genes that have already been linked to coronary disease.

**Conclusion:**

In conclusion, this study presents new genetic factors associated with severe stenosis and highlights different risk factors associated with a young age at diagnosis of CAD.

**Supplementary Information:**

The online version contains supplementary material available at 10.1186/s12920-021-00942-x.

## Background

Coronary artery disease (CAD) is defined as a multifactorial condition that results from complex interactions of modifiable risk factors and unmodifiable genetic determinants [1, 2]. Several risk factors have been determined to be commonly associated with CAD, such as age, sex, hypertension, type 2 diabetes, family history, and high cholesterol levels [3, 4].

According to the World Health Organization, noncommunicable diseases are a major cause of death in Lebanon with cardiovascular diseases (CVDs) accounting for 47% of the mortality rates [5]. The Middle East exhibits one of the highest CVD-associated mortality rates in the world [6]; and several studies, that have investigated the prevalence of the CAD associated risk factors, have drawn attention to the fact that prevalence is following an alarming increase over time [7].

Family and twin studies estimated that heritability of CAD ranges between 40 and 60% [8]. One loci (*9p21*) was shown to be associated with CAD and was associated with a 30% increased risk per copy of the risk allele [9–12]. Additional loci have been identified in different populations and mainly of European descent, via analyses on groups with significantly larger sample sizes [13, 14]. In 2015, the CARDIoGRAMplusC4D Consortium published a genome wide association study (GWAS) meta-analysis of 185,000 CAD cases and controls. This analysis investigated 6.7 million common variants as well as 2.7 million low-frequency variants and identified 10 novel loci associated with CAD [15].

Our study aims at comparing a group of patients having more than 50% stenosis in any coronary vessels to control subjects with no angiographic evidence of stenosis in these vessels. By studying the two extremes of this condition, we target to unravel strong genetic and environmental contributors to CAD. Our study revisits the significance of the previously mentioned risk factors of CAD in the Lebanese population and explores novel genomic loci.

## Methods

### Patient description

Patients were recruited at two major hospitals in Lebanon, between May 2007 and June 2010 as part of a multi-center cross-sectional study for the FGENTCARD Consortium (https://cordis.europa.eu/project/rcn/85024/factsheet/en) [16]. The degree of stenosis in selected coronary arteries was assessed by attending cardiologists and assigned a score as a percentage. In all the sample population, vessels were visualized and evaluated by angiography, namely the left main coronary artery, the left anterior descending artery, the right coronary artery, and the circumflex artery. The results from the angiography lead to the classification of the individuals depending on the level of stenosis in the visualized coronary artery. The date of the first workup cardiac catheterization, coronary artery bypass graft (CABG) and/or percutaneous transluminal coronary angioplasty (PTCA) was used to determine the age at CAD diagnosis.

Cardiologists performing the coronary angiography collected a 20 mL blood sample from the peripheral arterial access site of patients. Genomic DNA was extracted using a standard phenol chloroform extraction procedure. Trained healthcare professionals collected further data on the socio-demographic background of all patients (Additional file 1).

The epidemiological factors were determined from patient’s charts or status at time of enrollment. Positive smoking was defined as smoking status of the patient at the time of enrollment. Type 2 diabetes mellitus (T2DM) was mainly based on the patient medical records and confirmed with medication intake, such as insulin or oral hypoglycemic drugs, at time of enrollment. Hypertension and hyperlipidemia were also diagnosed by a physician according to guidelines at the time of presentation and confirmed by the prescription of anti-hypertensive and hypolipidemic drugs earlier to the time of enrollment.

Consanguinity was evaluated with three categories of relationship. The first category (c1) represented individuals whose parents are first cousins, the second category (c2) represented individual with parents being second cousins, and the third consanguineous category (c3) represented individuals with parents having a more distant relationship. In addition, family history of CAD and consanguinity status were combined in four different groups to evaluate the age at diagnosis in different combinations of these two factors. The groups were generated as follow: ‘f0c0’ representing patients with no evident family history nor consanguinity, ‘f0c1’ in which patients with first-cousin consanguinity but no family history were grouped, ‘f1c0’ for patients with family history of CAD but no significant consanguinity, and finally ‘f1c1’ for patients with both family history and first-cousin consanguinity.

### Study population

The initial cohort consists of 7710 individuals. In this study however, we included 2643 individuals that corresponded to the phenotypic selection of severe CAD and controls and on whom GWAS data was available. Previous analyses on the population showed consistency in the associations across the population stratifications with a possible increase in variability due to the small sample size [12].

### Selection of cases and controls

Subjects were assigned as cases or controls according to their stenosis levels. Cases were patients with at least 50% obstruction in any of the coronary arteries visualized and controls were patients with no stenosis [17]. Out of the 2643 subjects, we identified 1734 patients as cases and 757 subjects as controls making a total study population of 2491.

### Statistical analysis

The association of the different variables with the disease was evaluated by Pearson chi-square test adjusted through logistic regression and the comparison of means was done through independent t-tests, with significance threshold of *p* < 0.05. For every binomial variable, the risk estimate was evaluated by odds ratio (OR) with 95% confidence intervals. The adjustment of odds ratio in the logistic regression was done for standard risk factors for coronary artery diseases including age, sex, BMI, type 2 diabetes mellitus, hypertension, hyperlipidemia, smoking and family history of CAD, that were previously established to be related to CAD and young CAD [18]. The generation of all means, frequencies, and standard deviations was done using IBM-SPSS [19].

### Genome wide association study

DNA from the study population was genotyped by Illumina Human610-Quad BeadChip or Illumina Human660W-Quad BeadChip and the genotype data was pruned in PLINK [20, 21] for selection of valid genomes and autosomal regions. A total of 1745 subjects with more than 97% success genotyping rate were used for the analyses.

Three different models were used in this analysis using different combinations of factors previously proven significant in the study population. Three different models were evaluated with ‘snpStats’ [22] package in R 3.5.3 [23].

Model #1 includes the basic epidemiological determinants for coronary artery disease: sex, T2DM, hyperlipidemia, and hypertension.

Model #2 includes all factors included in Model #1 in addition to family history of CAD. Family history (Fx) was established for the patients if any first- or second-degree family member was clinically diagnosed with stenosis in coronary arteries.

Model #3 includes all the factors included in Model #2 in addition to Young age at diagnosis. Young age at diagnosis of CAD (YADCAD) was added as a variable to the population and was assigned to patients that were diagnosed at an age one standard deviation (SD) lower than the mean age of the population [18].

### SNP identification and annotation

The R package ‘qqman’ was used to plot the *p* values generated from the generalized linear model (GLM) representing the association of SNPs with extreme stenosis [24]. The Manhattan Plot was annotated with a *p* value of $5 \times 10^{ - 7}$ for significance threshold for the autosomes only. The Quantile–Quantile plot was generated to show appreciable deviation from the expected logarithmic *p* values for the significant nucleotide variations in the three different models.

The list of annotated SNPs that were reported to be significant were crossed with several genome databases such as the Human GRCh38/hg18 from the University of California, Santa Cruz [25, 26] and the “1000 Genomes Browser” from the National Center for Biotechnology Information [27, 28]. Results were reported based on the most recent version of the genome database: Genome Reference Consortium Human Genome build 38 [29].

### Linkage disequilibrium calculation

The evaluation of linkage disequilibrium (LD) for the most significant SNPs in the association models was done through the LD link application from the National Institute of Health [30]. Both r^2^ and D’ were extracted for SNPs in pairs in the European (EUR) population of the database including Utah Residents from North and West Europe (CEU), Tuscans in Italy (TSI), Finnish in Finland (FIN), British in England and Scotland (GBR), and Iberian population in Spain (IBS).

LD was calculated between the three significant SNPs from association models 1 and 2 and one variant previously associated with CAD in the Lebanese population.

## Results

### Population and subgroup descriptive statistics

Our study consisted of 2491 participants (Additional file 2) with 757 (30.3%) individuals having no stenosis and 1734 (69.5%) diagnosed with more than 50% stenosis in at least one of their major coronary arteries. A total of 254 (14.6%) affected individuals were categorized as young for CAD diagnosis, with a mean age of 44.4 years (± 4 0.4) compared to 64.2 years (± 8.8) for affected patients older than 49 years old, the latter being the threshold between the two categories.

**Fig. 1:**
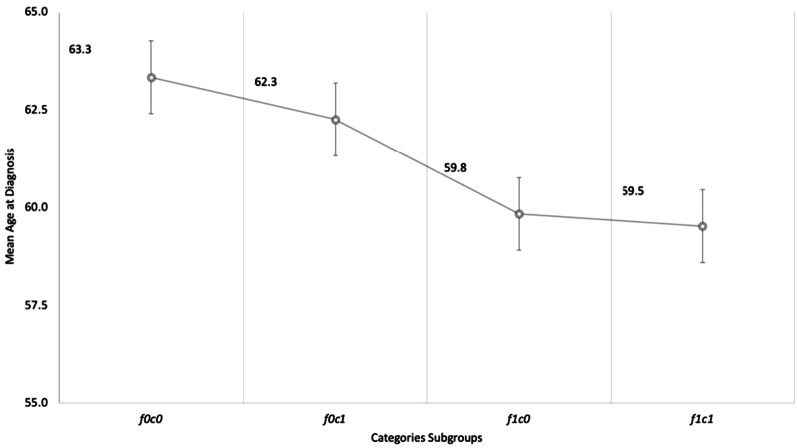
Trend of average age at diagnosis for four selected subgroups of CAD extreme patients with more than 50% stenosis with standard error bars for each subgroup average. f0c0, subgroup no FxCAD and no direct parental consanguinity; f0c1, no FxCAD but with direct parental consanguinity; f1c0, individuals with FxCAD and no parental consanguinity; f1c1, individuals with FxCAD and parental consanguinity

The overall mean age of onset in severe CAD patients was 60.9 (± 11.1) years compared with the control population mean age of 57.6 (± 11.5) year with a significance of $p = 4.5 \times 10^{ - 7}$. Within the affected population, the mean age at diagnosis for subgroup 1 ‘f0c0’ was 63.3 years, compared to 62.2 years for subgroup 2 ‘f0c1’, 59.8 years for subgroup 3 ‘f1c0’ and 59.5 years for subgroup 4 ‘f1c1’. Even though the difference in the age of onset between the four subgroups did not reach statistical significance, the mean age of onset decreased across positive family history and consanguinity subgroups (Fig. 1). This trend shows a probable correlation of the age at diagnosis with positive family history and consanguinity.

Family history shows a significant association with the disease phenotype with a *p *value of 0.002 and an OR of 1.4 (CI = [1.1–1.8]). In addition, 64% of the general population identified a direct family member with CAD and 71.5% of the affected population identified as having a family history of CAD (Table 1). The direct effect of consanguinity was not statistically significant (*p* = 0.43). This might be due to the fact that consanguinity was hard to evaluate in our population because of the social bias (Table 2).

**Table 1:** Adjusted summary statistics of epidemiologic and diagnostic factors in controls and CAD extremes patients

Variables	CAD categories				*p* value	OR (*SD*)	Confidence interval (95%)	Total No
	Category 1		Category 2					
	No	% (*SD*)	No	% (*SD*)				
Mean age at diagnosis, in years (SD)	58	(*11.5*)	61	(*11.1*)	< 0.001	(*0.7*)	[2.1–4.7]	2493
Female	58	(*10.6*)	65	(*10.3*)		(*0.8*)	[4.7–7.9]	
Male	54	(*14.4*)	60	(*11.1*)		(*1.8*)	[1.8–9.1]	
Sex					< 0.001	3.0	[2.4–3.7]	2491
Female	356	44.4	445	55.6				801
Male	401	23.7	1289	76.3				1691
Diagnosed diabetic					< 0.001	2.7	[2.1–3.4]	2488
No	618	35.9	1102	64.1				1720
Yes	137	17.8	631	82.2				768
Diagnosed hypertensive					< 0.001	2.0	[1.6–2.5]	2488
No	379	38.2	613	61.8				992
Yes	376	25.1	1120	74.9				1496
Diagnosed hyperlipidemic					< 0.001	1.6	[1.3–1.9]	2486
No	463	35.8	832	64.2				1295
Yes	291	24.4	900	75.6				1191
Family history of CAD					0.002	1.4	[1.1–1.8]	2485
No	315	33.8	617	66.2				932
Yes	442	28.5	1111	71.5				1533

For the age, the values reported are the mean age for each category followed by their respective standard deviation (SD) reported in italic

Odds ratios were adjusted for age, sex, hypertension, hyperlipidemia, type 2 diabetes, BMI, smoking and family history

CAD categories:

Category 1: individuals with 0% stenosis in any major coronary vessel

Category 2: individuals with more than 50% stenosis in any major coronary vessel

**Table 2:** Summary statistics of the consanguinity factor on the overall population

Ancestry	CAD categories			
	Category 1		Category 2	
	No. (within categories)	% (within categories)	No. (within categories)	% (within categories)
No consanguinity	541	30.1	1225	69.3
Consanguinity	169	31.2	372	68.8
First cousins	*(110)*	*(65.1)*	*(229)*	*(61.5)*
Second cousins	*(36)*	*(21.1)*	*(49)*	*(13.2)*
More	*(23)*	*(13.8)*	*(94)*	*(25.3)*

The percentages are representative of the overall CAD categories and within each category for positive consanguinity factor, the values within positive consanguinity were reported as first, second or farther cousins (are represented in italic)

Hypertension had a very strong association with the phenotype of the patients with a *p *value $< 0.001$ and odds ratio of 2.0 (CI = [1.6–2.5]) (Table 1). A total of 60.1% of the study population was diagnosed with clinical hypertension, of which, 74.9% presented with CAD. T2DM is less common in our sample population compared to hypertension, with 31% of the population diagnosed with T2DM, of whom 82.2% are diagnosed with CAD (Table 1). The positive association of T2DM with the extreme CAD phenotypes is significant with a *p *value < 0.001 with an odds-ratio of 2.1 (CI = [2.1–3.4]). Hyperlipidemia also shows a significant positive correlation with CAD, as 75.6% of the hyperlipidemic individuals diagnosed with severe stenosis with a *p *value < 0.001 and an odds ratio of 1.6 (CI = [1.3–2.9]) (Table 1).

In addition to standard epidemiological factors evaluated in CAD, we considered the young age at diagnosis as another factor of stratification. This additional filtering aims at identifying factors that play a role in the earlier expression of the disease. The results yielded a significant correlation of the disease with smoking. The mean age at diagnosis for the young cases was 44.4 (± 4.41) and was significantly lower than the general severe CAD population (*p* < 0.05). In the YADCAD, 74% had a positive current smoking status, with a *p-*value of 8.07 × 10^–10^ (Table 3). T2DM and hypertension showed a negative correlation with this group (Table 3). More than 70% of the YADCAD population had no history of diabetes $\left( {p = 0.00038} \right)$. In addition, hypertension had the same lack of correlation with the young diagnosed population with only 43.9% of this population being diagnosed as hypertensive ($p = 7.9 \times 10^{ - 14}$) (Table 3). In contrast, family history showed a significant positive correlation with this category of the affected population with 77.6% of the YADCAD population having a positive history of family CAD $(p = 0.000002)$ (Table 3).

**Table 3:** Summary statistics of the young age at CAD diagnosis in the sample population

Variables	Young age at diagnosis (YADCAD)				*p* value	OR	Confidence interval (95%)	Total No
	No		Yes					
	No	% within YADCAD	No	% within YADCAD				
Family history of CAD					< 0.001	2.1	[1.5, 2.9]	
No	560	*38.0*	57	*22.4*				617
Yes	914	*62.0*	197	*77.6*				1111
Diagnosed diabetic					< 0.001	0.6	[0.4, 0.8]	
No	916	*61.7*	186	*73.5*				1102
Yes	567	*38.2*	67	*26.5*				634
Diagnosed hypertensive					< 0.001	0.4	[0.3, 0.5]	
No	471	*31.8*	142	*56.1*				613
Yes	1009	*68.2*	111	*43.9*				1120
Smoking					< 0.001	3.1	[2.2, 4.5]	
Never smoked	494	*52.2*	45	*26.0*				586
Current Smoker	451	*47.8*	128	*74.0*				579

No. represent the number incidence for specific traits

The percentages in italics are indicative within the YADCAD or non-YADCAD population

### Genome wide association studies

For the standard association, a *p* value of $5 \times 10^{ - 5}$ was considered as the threshold to compare with previous studies on coronary artery disease (Reported in the Additional file 3). The list of SNPs significant for this threshold was used and compared to significant SNPs for CAD reported recently in the literature for the Lebanese population [31]. Six out of twenty SNPs reported previously in the original population were found in the Model 0 [31].

**Fig. 2:**
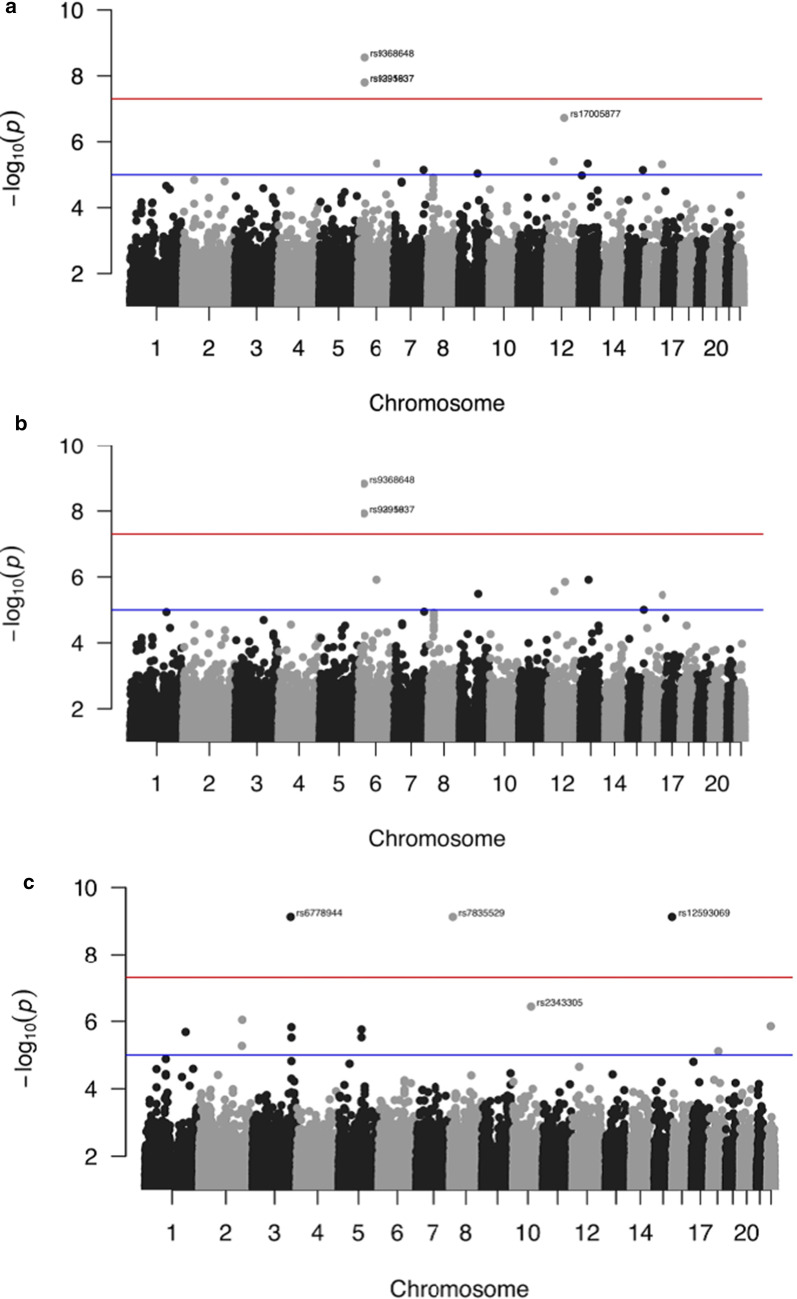
**a** Manhattan plot for the first model of linear regression, with annotation of the SNPs that are above the selected threshold for the *p* value $\left( {5 \times 10^{ - 7} } \right)$ represented as a black line. *rs9391637 and rs9295937 overlap on the plot.* **b** Manhattan plot for the second model of linear regression, with annotation of the SNPs that are above the selected threshold for the *p* value $\left( {5 \times 10^{ - 7} } \right)$ represented as a black line. *rs9391637 and rs9295937 overlap on the plot.* **c** Manhattan plot for the third model of linear regression, with annotation of the SNPs that are above the selected threshold for the *p* value $\left( {5 \times 10^{ - 7} } \right)$ represented as a black line

**Fig. 3:**
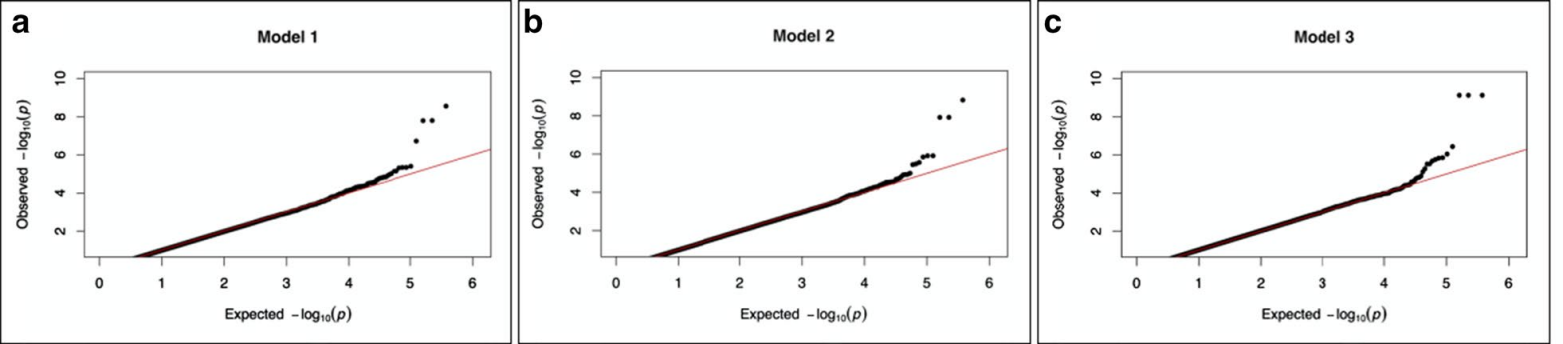
**a** Quantile–Quantile (QQ) plot for the first model of linear regression, showing the variation in the Expected versus Observed *p* value for the studied SNPs. **b** Quantile–Quantile (QQ) plot for the second model of linear regression, showing the variation in the Expected versus Observed *p* value for the studied SNPs. **c** Quantile–Quantile (QQ) plot for the third model of linear regression, showing the variation in the Expected versus Observed *p* value for the studied SNPs

The threshold used for genome-wide significance was $p = 5 \times 10^{ - 7}$ for the three different models. In the first regression model for the severe CAD phenotype, which accounts for sex, T2DM, hyperlipidemia, and hypertension as cofactors, we were able to identify a total of four SNPs on two different chromosomes: rs9368648 (*p* = $2.765286 \times 10^{ - 9}$), rs9391637 (*p* = $1.598328 \times 10^{ - 8} )$, and rs9295937 (*p* = $1.576084 \times 10^{ - 8} )$ on chromosome 6; rs17005877 (*p* = $1.886510 \times 10^{ - 7} )$ on chromosome 12 (Table 4, Figs. 2a, 3a). In the second model with family history of CAD as an added cofactor, we identified three SNPs on chromosome 6: rs9368648 (*p* = $1.483080 \times 10^{ - 9}$), rs9391637 (*p* = $1.189551 \times 10^{ - 8} )$, and rs9295937 (*p* = $1.183131 \times 10^{ - 8} )$(Table 4, Figs. 2b, 3b). As for the third model which adds YADCAD, we were able to identify four SNPs on four different chromosomes: rs6778944 (*p* = $7.431757 \times 10^{ - 10}$) on chromosome 3, rs7835529 (*p* = $7.445635 \times 10^{ - 10}$) on chromosome 8, rs2343305 (*p* = $3.660188 \times 10^{ - 7} )$ on chromosome 10, and rs12593069 (*p* = $7.433340 \times 10^{ - 10} )$ on chromosome 15 (Table 4, Figs. 2c, 3c).

**Table 4:** Summary table for the SNPs significant for each of the Generalized Linear Models (GLMs)

Lead SNP	Position	Chromosome	A1	A2	Candidate genes	Variant location	*p* value for association in every model		
							Model #1	Model #2	Model #3
rs17005877	*79763772*	12	G	A	N/A	Intergenic	1.89E−07	N/S	N/S
rs9295937	*30961632*	6	G	A	*NAPGP2*	Pseudogene	1.58 E−08	1.18 E−08	N/S
rs9368648	*30956152*	6	T	C	N/A	Intergenic	2.77 E−09	1.48 E−09	N/S
rs9391637	*30945130*	6	G	A	*MUCL3*	Intronic	1.60 E−08	1.19 E−08	N/S
rs12593069	*101355666*	15	C	T	*PCSK6*	Intronic	N/S	N/S	7.43 E−10
rs2343305	*80468431*	10	A	G	*TSPAN14*	Intronic	N/S	N/S	3.66 E−07
rs6778944	*171548961*	3	G	T	N/A	Intergenic	N/S	N/S	7.43 E−10
rs7835529	*13664494*	8	T	C/G	N/A	Intergenic	N/S	N/S	7.45 E−10

The significance level was set at $5 \times 10^{ - 7}$ for each of the three models and the nucleotide locations and annotation were based on the human genome reference GRCh38/hg18.

### Linkage disequilibrium

In the first model, three of the reported SNPs are present on the same chromosome: rs9368648, rs9391637, and rs9295937. LD was calculated in pairs and for all three pairs the r^2^ and the D’ were equal to 1. The remaining of the SNPs reported significant in the three models are not on the same chromosome and thus LD was not studied (Table 5).

**Table 5:** Summary table for four LD pairs of SNPs on chromosome 6

SNP pair		D’	r^2^	In phase alleles
rs9391637	rs9295937	1	1	AA/GG
rs9368648	rs9295937	1	1	CA/TG
rs9391637	rs9368648	1	1	AC/GT
rs9368648	rs9349379	1	0.00074	CA/TG
rs9391637	rs9349379	1	0.00124	AA/GG
rs9295937	rs9349379	1	0.00124	AA/GG

SNPs rs9391637, rs9368648, and rs9295937 were shown to be significant in our results. *PHACTR1* specific SNP (rs9349379) was evaluated for LD

LD was also calculated for the three above mentioned SNPs with a specific locus from the *PHACTR1* gene that was previously recently reported in association with CAD phenotype in the Lebanese population, rs9349379 [32]. The D′ in all the three cases were reported as 1, even though the r^2^ values were low. For the LD with rs9368648, rs9391637, and rs9295937, the r^2^ values were 0.000743385, 0.00124474 and 0.00124341 respectively (Table 5).

## Discussion

In this study, we evaluate the severe CAD phenotype in the Lebanese population with its unique characteristics and genetic heritage [33]. Our results deviated from previous reports of CAD in the Lebanese population. This deviation likely results from the different and more stringent criteria employed for the selection of cases and controls as well as the various models used in the genome wide association analyses.

The present study population included patients that were referred to cardiovascular consult for different presentations and were assessed in different hospitals in Lebanon. This variability in clinical diagnosis could be considered as a limitation. In addition to that, atherosclerosis was considered as the cause of coronary vessel stenosis, this could also be considered as a limitation of the study. More than that, the association of consanguinity was not completely evaluated, as some population bias may have interfered the reporting of this variable.

The standard epidemiological determinants of CAD showed significant correlation in our population. In addition, the population stratification by the positive/negative consanguinity and family history factor showed a trend of correlation with the age at diagnosis. Although the difference between the mean ages of the four categories is not statistically significant, there was a sequential age decrease from the category with no family history or consanguinity to the category positive for both conditions. This stratified trend is relevant to the Lebanese population where consanguinity is considered high compared to other societies [32].

The GWAS association of the first two models showed genome wide significant CAD susceptibility SNPs common for both models. These SNPs had not been reported in the CAD literature. The most significant SNP in both models, rs9368648, is an intergenic variant on chromosome 6. This variant is 2 kb downstream from *HCG21*. *HCG21* is responsible for the expression of human leukocyte antigen 21 (HLA) that has been linked to the inflammatory process involved in coronary disease and other vascular diseases. The second most significant variation, rs9391637, on chromosome 6 is an intronic variant of *MUCL3*, responsible for the expression of MUCL3 protein and acts as an enhancer for genes related to CAD and mean platelet volume with no definitive linkage to the diseases [34]. Rs2925937 is the third significant variation for both models 1 and 2. This polymorphism is on chromosome 6, in the pseudogene NAPGP2. This region of the genome has already been linked to congenital heart defects and conotruncal heart defect [35], which are related to abnormalities in the outflow of blood from the heart, and may play a significant role in the blood flow of major vessels [36]. The three abovementioned SNPs are also in strong linkage, which reinforces the importance of this region of chromosome 6 in coronary disease. Within this region, seven CAD variants have been previously associated to CAD, of which *PHACTR1* that has been associated go CAD in the Lebanese population in a prior study [13, 37]. Linkage disequilibrium between the three most significant SNPs in this study along with the point variant previously reported in *PHACTR1* generated low r^2^ values. These low values underscore the intergenic population variabilities and the need to have susceptibility alleles studies independently replicated in numerous populations [38].

Rs17005877, is an intergenic variant of chromosome 12 that was only significant in the first model. It is located between *PAWR* and *PPP1R12A* genes and its association with CAD has never been reported before. PAWR, located 72.6 kb upstream of rs17005877, is responsible for the regulation WT1 pro-apoptotic pathway involved in cardiovascular differentiation and disease [39]. PPP1R12A, located 9.7 kb downstream of rs17005877, is involved in the expression of light-chain myosin phosphatase. This protein belongs to the family of myosin targeting subunit (MYPT), which is involved in the regulation of light chain myosin phosphatase recently implicated in the aberrant contractility associated with atherosclerosis [40–42].

The young age at diagnosis was used to stratify the patient population and to investigate the factors that may have a link with early onset CAD. The third model identified four novel CAD susceptibility SNPs. The first, rs6778944 is an intergenic variant on chromosome 3, 44 kb away from a pseudo-gene, *RNU6-348P*, not previously reported in CAD patients. The second, rs12593069 on chromosome 15, is intronic to *PCSK6*. *PCSK6* is involved in lipid metabolism and variations in this gene have been previously associated with atherosclerosis [43]. *PCSK9*, another gene of the same family, has been reported in several GWAS as significantly associated with hyper-LDL-cholesterolemia in CAD [13, 37, 43]. The third SNP, rs785529 on chromosome 8, is 60 kb away from *DLC1*, a gene responsible for Rho-GTPase activity and has a high expression in fat tissues [44]. DLC1 was recently linked to congenital heart disease in the Chinese population [45]. In addition, the general functions of Rho-GTPase are closely related to cardiovascular disease in the context of vessel contraction, oxidative stress, and inflammation and is being targeted as a potential treatment for the general cardiovascular disease [46, 47]. The last significant SNP in our model, rs2343305 on chromosome 10, is an intronic variant of *TSPAN14*. This tetraspanin gene has its highest expression in fat tissues and is related to the platelet interaction with endothelial cells. It may interact with the inflammatory pathway of atherosclerosis, which has a major role in CAD, but was never previously reported as directly associated with the disease [44, 48].

In our population, stratification validated the importance of YADCAD and helped us elaborate on the uniqueness of this stratification showing distinctive epidemiological factors and loci for sever CAD. The replication of the association with different factors showed variations in the genes correlated to the severe phenotype of the disease.

Overall, the significant SNPs for severe CAD in young population analysis revealed new loci related to the disease, some of which were part of gene families already mentioned in the CAD literature namely lipid metabolism, atherosclerosis-related inflammation.

## Conclusion

This study evaluated the difference in affected CAD population by the age of onset both in epidemiological determinants and in genome-wide variations. Young age CAD patients should be considered separately as epidemiological determinants for the disease vary and even genomic relations differ. Continued exploration of clinical presentations and genetic variations is required to understand better the distinctiveness of young age CAD, and the public health community should adapt treatment and prevention to younger populations and their specific risk factors compared to the general population.

## Supplementary Information

**Additional file 1.** The survey that was used to record the data at time of patient interview.

**Additional file 2.** The title is “Characteristics of Sample population”. The table includes several epidemiological factors included in the present paper and reported as individual tables, but it also includes descriptive of the population that can be viewed as useful by readers to have a better understanding of the sample. These include means and standard deviations for BMI and Total cholesterol levels in both controls and severe CAD patients, reason for patient catheterization and finally the percentages of cardiovascular comorbidities in both populations. All the descriptive were reported as absolute numbers as well as percentages within controls and within CAD population respectively.

**Additional file 3.** The title is “List of SNPs for regression models significant for p-value below 0.00005”. These results include all the list of SNPs for all three models of associations that have a significance level below the above-mentioned threshold. This information was added to the file directory of the present paper to have a more objective approach and compare objectively our results to previous reports on the population.

## Data Availability

The majority of the data analyzed is included in the article or as additional files. The significant datasets generated and/or analyzed during the current study are available in the NCBI dbSNP repository with the following submission reference: SUB9162284*.*

## Abbreviations

BMI: Body mass index
CABG: Coronary artery bypass graft
CAD: Coronary artery disease
CI: Confidence interval
CVD: Cardiovascular disease
FxCAD: Family history of coronary artery disease
GLM: Generalized linear model
GWAS: Genome-wide association study
LD: Linkage disequilibrium
OR: Odds ratio
PTCA: Percutaneous transluminal coronary angioplasty
SD: Standard deviation
SNP: Single nucleotide polymorphism
T2DM: Type 2 diabetes mellitus
YADCAD: Young age at coronary artery disease diagnosis
