# Supplementary material for: New susceptibility alleles associated with severe coronary artery stenosis in the Lebanese population
Source: BMC Med Genomics. 2021 Mar 25;14:90. doi: 10.1186/s12920-021-00942-x (PMC7993530; doi:10.1186/s12920-021-00942-x)
Supplement: Supplementary file 1 — Additional file 1. The survey that was used to record the data at time of patient interview. [file 12920_2021_942_MOESM1_ESM.pdf]

Lebanese American University
School of Medicine

Cardiovascular Database

ID # ______

General Information:

Age:	Race: White ☐ Black ☐ Asian ☐
Sex: M☐ F☐	Religion:
Admitting Physician:	Origin and Place of Living:
Admission Date:	Occupation:
Other Hx. of CV Admission: Yes☐ No☐	Birth Weight: Weight: Height:
Gestational age: Preterm ☐ full term☐ Posterm☐	Maternal pregnancy complications - Yes☐ No ☐

Female Reproductive Health:

Female Menarche age<br>Menopause age<br>Menses hx(regularity) Y N Hirsutism Hx	Married ☐<br>Single ☐	Gravida<br>Para	Miscarriage<br>Abortion	Age first/last Gravida
OCP: Date Duration	Breastfeeding duration:			
HRT: Date Duration	Infertility Hx: Y N Rx: po IVF			

Health Behavior:

**Diet:**<br>Regular ☐<br>Weight-Control ☐<br>Low Salt ☐<br>Diabetic ☐<br>Low Animal Fat ☐<br>Uremic(Low Protein) ☐<br>Other ______	**Smoking:**<br>Yes ☐ No ☐ ___PY
**Activity Level:**<br>Physically Inactive ☐<br>Moderately Active ☐<br>Athletic ☐	**Caffeine Consumption:**<br>None ☐<br>1-2 cups/d ☐ 3-5 cups/d ☐ >5 cups/d ☐
**Stress Level:**<br>Low ☐ Moderate ☐ High ☐	**Alcohol Consumption:**<br>None ☐<br>Occasional ☐ 1 drink/d☐ > 1 drink/d☐

Family Hx.:

Cardiac Disease	Yes☐ No☐<br>Age < 55yrs.☐ Age >55 yrs.☐
Hypertension	Yes☐ No☐
Diabetes Mellitus	Yes☐ No☐
Hyperlipidemia	Yes☐ No☐
Consanguinity in parents	Yes☐ No☐ Specify:

Committee on Human Subjects in Research
Lebanese American University
13 DEC 2007
APPROVED

Version 3
Feb, 2006

ID # ________

Medical Hx

Hypertension	Yes☐	No☐	Renal Disease	Yes☐	No☐
Diabetes Mellitus	Yes☐	No☐	Rheumatic Fever	Yes☐	No☐
Hyperlipidemia	Yes☐	No☐	SLE	Yes☐	No☐
Menopause	Yes☐	No☐	Polycythemia	Yes☐	No☐
Sickle-cell anemia	Yes☐	No☐			

Previously Prescribed Medications:

Nitrates ☐	ACE inhibitors ☐	β-blockers ☐	Antiplatelets ☐
Inotropes ☐	Ang II R blockers ☐	Ca^{+2}-channel blockers ☐	Anticoagulants ☐
Diuretics ☐	Centrally-acting antiHTN☐	Antiarrhythmics ☐	Thrombolytics ☐
Cardiac Glycosides☐	α_1-blockers ☐		
	Other Vasodilators ☐		

Cardiovascular Diagnostic Tests:

Noninvasive: Test	Result		Date	Time
**Echo**	EF<45% (___) 45 %<EF<55%☐	EF>55%☐		
**Treadmill**	ST changes	no ST changes ☐		
**Thallium Scan**	Ischemia Inf ☐	Infarction Inf ☐		
	Ant ☐	Ant ☐		
	Lat ☐	Lat ☐		
**EBT- Calcium scoring**				
**Holter**				
**Other**				

Committee on Human Subjects in Research
Lebanese American University
13 DEC 2007
APPROVED

Invasive:	Test	Result			Date	Time
	**Cardiac Cath.**	LAD <50%	50-70%	>70%		
		RCA <50%	50-70%	>70%		
		Cx <50%	50-70%	>70%		
	**PTCA**	Yes☐ No☐	**Stent insertion**	Yes☐ No☐		
		LAD ☐		LAD ☐		
		RCA ☐		RCA ☐		
		Cx ☐		Cx ☐		
	**EP Studies**					
	**Other**					

Heart Rate: **Date**

Blood Pressure: **Date:**

ID # ________

Clinical Diagnosis:

Stable Angina ☐	Myocardial Infarction ☐
Unstable Angina ☐	CAD ☐

CHF ☐	Cardiogenic Shock ☐	Pulmonary HTN ☐	Cor Pulmonale ☐
Pulmonary Edema ☐	Cardiac Tamponade ☐	Pulmonary Embolism ☐	

Atrial Fibrillation ☐	PVCs ☐	1° AVB ☐
Atrial Flutter ☐	Ventricular Tachycardia ☐	2° AVB ☐
SVT ☐	Ventricular Fibrillation ☐	AV Dissociation ☐
	Asystole ☐	Others ☐

Aortic Aneurysm ☐	Hypertension ☐	Digitalis Toxicity ☐
Aortic Dissection ☐	Hypotension ☐	

Pericarditis ☐	Myocarditis
Endocarditis ☐	

Mitral Valve Prolapse	Tricuspid Valve Regurge
Mitral Valve Regurge	Tricuspid Valve Stenosis
Mitral Valve Stenosis	Pulmonic Valve Regurge
Aortic Valve Regurge	Pulmonic Valve Stenosis
Aortic Valve Stenosis	Cardiomyopathy
Congenital Heart Disease	Cardiac Tumor

Others:	

Committee on Human Subjects in Research
Lebanese American University
13 DEC 2007
APPROVED

Version 3
Feb, 2006

Clinical Interventions:

Pharmacological:

Nitrates ☐	ACE inhibitors ☐	β-blockers ☐	Antiplatelets ☐
Inotropes ☐	Ang II R blockers ☐	Ca^{+2}-channel blockers ☐	Anticoagulants ☐
Diuretics ☐	Centrally-acting antiHTN☐	Antiarrhythmics ☐	Thrombolytics ☐
Cardiac Glycosides☐	α_1-blockers ☐		
	Other Vasodilators ☐		
Antibiotics ☐			

Other Noninvasive Intervention:

Cardioversion ☐ Defibrillation ☐

Surgical: CABG ☐ MVR ☐ AVR ☐ Other ☐

Other Invasive Intervention:

Pacemaker insertion ☐ AICD insertion ☐

IABP ☐ Drainage of Cardiac Temponade ☐ Endotracheal intubation☐ Other☐

ID#________

Biochemical Profile:

Genetic Tests:

DNA

Metabolic Factors: Results Date Time

Lipid Profile:

Total Cholesterol (mg/dl) HDL (mg/dl) LDL (mg/dl) TG (mg/dl)

Cardiac Enzymes: Date

LDH (IU/L) CPK (IU/L) CPK-MB (IU/L) Troponin T

Time 1

Time 2

Time 3

C-Reactive Protein mg/dl

Homocysteine Levels micromol/l

Glucose

Uric Acid

LP(a)

Pro-BNP

Haematological Factors:

Committee on Human Subjects in Research
Lebanese American University
13 DEC 2007
APPROVED

Creatinine:
GFR:

CBC:

Other lab tests;
